# Predictors of missed HIV screening opportunities among newly diagnosed individuals at an urban medical center in New York City, 2018–2022

**DOI:** 10.1371/journal.pone.0290414

**Published:** 2023-09-07

**Authors:** Jeffrey Paer, Judy Ratcliffe, Michelle Chang, Caroline Carnevale, Daniela Quigee, Peter Gordon, Susan Olender, Magdalena E. Sobieszczyk, Jason Zucker

**Affiliations:** 1 Department of Medicine, Columbia University Medical Center, New York, New York, United States of America; 2 Division of Infectious Diseases, Department of Medicine, Columbia University Medical Center, New York, New York, United States of America; 3 Division of Infectious Diseases, Department of Medicine and Pediatrics, Columbia University Medical Center, New York, New York, United States of America; University of New South Wales, AUSTRALIA

## Abstract

**Objective:**

To identify demographic and clinical factors predictive of having a missed opportunity (MO) for HIV screening.

**Design:**

Retrospective cohort study.

**Methods:**

Electronic medical records were queried for individuals newly diagnosed with HIV in different sites within a large urban academic medical center in New York City between 2018 and 2022. The primary outcome was having one or more MO for HIV screening within the institution, defined as any encounter at which screening was not performed in the 365 days preceding the HIV diagnosis.

**Results:**

Over one third of new diagnoses had at least one MO in the preceding year. Older individuals, cisgender women and those assigned female sex at birth, and heterosexual individuals were more likely to have at least one MO. An initial CD4 < 200 cells/ul was more likely among men who have sex with women specifically. Most MOs occurred in the emergency department and outpatient settings, with minimal HIV prevention discussions documented during each MO.

**Conclusions:**

These findings suggest that populations perceived to be at lower risk for HIV are more likely to have MOs and possibly late diagnoses, and that universal HIV screening must be implemented into the workflows of emergency department and outpatient settings to facilitate early diagnosis and reduce the incidence of HIV.

## Introduction

In 2019, approximately 1.2 million people in the United States (US) were living with HIV, of which approximately 13% are believed to be undiagnosed. In 2020, 30,635 people in the US were newly diagnosed with HIV, though data is limited due to the COVID-19 pandemic. Of those newly diagnosed, transmission was predominantly male-to-male and disproportionately affected Black and Hispanic communities [1]. Importantly, individuals unaware of their HIV positive status account for approximately 40% of ongoing transmissions in the US [2]. Early diagnosis and immediate antiretroviral therapy initiation lead to reduced risk of transmission and disease progression, highlighting the importance of widespread HIV screening [2, 3].

The Northeast remained the region with the highest proportion of HIV per 100,000 people [1]. In 2014, New York State (NYS) created a plan to end the AIDS epidemic by 2020 known as Ending the Epidemic (ETE) blueprint. The ETE initiative sought to identify persons with undiagnosed HIV, engage and retain them in healthcare to maximize virologic suppression, and facilitate access for individuals without HIV to Pre-Exposure Prophylaxis (PrEP) [4]. In 2010, NYS mandated that all healthcare providers offer HIV screening to patients 13 years or older in hospital or primary care settings, though patients must be notified of HIV screening [5].

Despite this mandate, numerous patient, provider, and institutional factors contribute to suboptimal HIV screening rates [6]. Fear of interpersonal conflict or abandonment, stigma, privacy concerns, inconvenience (e.g., transportation), poor access to care, and low health literacy or perception of risk are common patient-level barriers [7]. Many provider-level barriers reflect a need for further training on routine, culturally competent HIV screening as an evidence-based practice that should be integrated into inpatient and outpatient workflows [8]. Prior studies have identified emergency departments (EDs) and primary care clinics as frequent sites of missed HIV screening opportunities [8–10].

A previous study completed at our institution examined missed opportunities for HIV testing (MOs) from 2006 to 2017 and found that 36% of patients newly diagnosed with HIV had at least one MO for HIV screening with no reduction in MOs over time [11]. Due to the COVID-19 pandemic, ETE efforts have been disrupted: HIV-related laboratory testing decreased by 66% in April 2020, while monthly reporting decreased by 24% when compared to 2019 and has not yet returned to pre-pandemic levels [12]. The total number of HIV tests distributed nationwide in health care settings in 2020 was reduced by almost half compared to 2019 [13]. NYS has now revised the ETE timeline to 2024 [12]. Additional barriers to HIV screening during this time period include health system limitations such as time constraints (both in ED and outpatient settings) and higher-priority medical issues; systemic disruptions to PrEP supply and distribution in the form of suspended outreach and reduced personnel also occurred [14, 15].

In this study, MOs for HIV screening were examined at an academic urban center between the years 2018 and 2022. Among our cohort of individuals newly diagnosed with HIV, we aimed to identify the demographic and clinical factors associated with MOs for HIV screening and factors associated with late diagnosis of HIV as defined by an initial CD4 <200 cells/ul. We also sought to determine the sites within our institution where MOs occurred most frequently.

## Materials and methods

This study was conducted at a large urban academic medical center comprising inpatient, outpatient, and emergency medical services at two main campuses in northern New York City. The medical center is located in a community in which 72% of residents identify as Hispanic, 25% of households are below the federal poverty line, and HIV prevalence is over 2% [16]. The medical center is a New York State Designated AIDS Center providing HIV/HCV care and prevention services in the Comprehensive Healthcare Program (CHP). For every positive HIV test result, the CHP is notified and assists with post-test counseling and linkage to care for newly diagnosed individuals.

All new HIV diagnoses between January 1, 2018 and June 30, 2022 were identified in the HIV CHP database. Medical records were queried for demographics, inpatient and outpatient visits, sexually transmitted infection screening, and HIV prevention discussions. New diagnoses were defined as patients of any age with positive HIV 4th generation screening test and confirmatory antibody tests (and/or detectable HIV viral loads) who did not have prior positive HIV screening upon chart review. Individuals with a positive 4th generation screening test done outside the institution who presented for confirmation were also considered new diagnoses. All data, including patients’ medical record numbers and dates of birth, were entered into a REDCap database. Each patient was given a unique study identification number. When the data was downloaded for REDCap for analysis, only the study identification number and date of birth, but not the medical record number, were included in the final dataset for analysis. All identifiers were removed after data collection was complete.

It is important to note that the sexual activity variable was comprised of gay and bisexual men who have sex with men (GBMSM), men who have sex with women (MSW) and women who have sex with men (WSM). All GBMSM were individuals who were assigned male sex at birth who reported having sex with cisgender men. Of the 143 total GBMSM, 129 were cisgender men, 4 were gender non-conforming, and 10 were transgender women. These individuals identifying as gender non-conforming and transgender female were included within the GBMSM category due to small sample sizes precluding the creation of the additional sub-variables of “gender non-conforming individuals assigned male sex at birth who have sex with men” and “transgender females who have sex with men.” In addition, of the 143 total GBMSM, 8 also had sex with cisgender women. There was one individual who self-identified as a transgender male who had sex with cisgender men who was not included in this analysis due to difficulty classifying his sexual behavior with these categories.

The primary outcome was having one or more MO for HIV screening within the institution, defined as in previous studies as an ED, inpatient, or outpatient visit at which screening was not performed in the 365 days preceding the HIV diagnosis [11, 17]. This definition of a MO was chosen as the CDC recommends annual screening in patients of increased risk [18]. Given our cohort of individuals newly diagnosed with HIV, every individual in this study should have been considered high risk and should have received at least one HIV screening test in the preceding year. Any encounters at other medical centers or clinics outside of the institution were excluded from the individual’s total count of MOs, even if clearly recorded or referenced in the medical record. As such, having zero MOs could indicate that the new HIV diagnosis occurred either (1) without any prior encounters within or outside the study site or (2) after having prior encounters outside the study site (eg, if an individual who newly acquired HIV presented to medical centers outside of the study site several times without testing but then had a positive HIV test during the first encounter at the study site, they would be categorized as having zero MOs). Given any encounters at other medical centers outside of the institution were excluded from the analysis, the proportion of individuals with zero MOs falling into the aforementioned first or second scenarios were not recorded in the analysis.

In a secondary analysis, new diagnoses were grouped into pre-COVID-19 and COVID-19 eras using the breakpoint of February 29, 2020, which was the date of the first confirmed case in New York City [19].

Descriptive statistics were used to compare demographic and clinical characteristics of new diagnoses with and without MOs. Univariable logistical regression, including multiple comparison correction, was performed to identify factors predictive of having at least one MO vs zero MOs as well as factors predictive of late diagnosis defined as a CD4 of less than 200 cells/ul at the time of the initial CD4 count within 6 months of the HIV diagnosis. Variables with p ≤ 0.2 rather than p ≤ 0.05 were included in the multivariable analysis to reduce omitted-variable bias. Due to co-linearity between sex assigned at birth, gender, and sexual activity, the multivariable regression included sexual activity but not sex assigned at birth or gender. In the regression, individuals were excluded if values for any variable were missing. All statistical analyses were performed with RStudio 2022.02.2, build 485.

This study was approved by the Columbia Human Protection Office Institutional Review Board (IRB-AAAS1368). This was a retrospective chart review study conducted with an IRB approval containing a waiver of informed consent. Although individuals newly diagnosed with HIV between January 1, 2018 and June 30, 2022 were included in the analysis the date range in which individual participants’ medical information (eg, prior sexually transmitted infection testing history) was collected for analysis was January 1, 2010 through June 30, 2022. Following completion of data collection, authors did not have access to any information that could identify individual participants other than dates of birth.

## Results

Between January 2018 and June 2022, there were a total of 260 new HIV diagnoses and 91 (35%) had at least one MO in the previous year. There were a total of 238 MOs, with a median of 2.0 (IQR 1.0–3.0) per person.

The majority of new diagnoses occurred in those who were 25–49 years of age (65%), male at birth (80%), and self-identifying as cisgender male (75%), Hispanic (49%) and non-Hispanic Black (42%).

In the univariable analysis, newly diagnosed individuals with at least one MO were more likely to be ≥50 years old compared with patients ≤25 years old (27% vs 11%; OR 3.15 (1.35–7.58) p = 0.003). Newly diagnosed individuals with at least one MO were also more likely to be sexually assigned female compared to male at birth (26% vs 16%, OR 1.97 (1.05–3.69), p = 0.035), identify as cisgender women compared to cisgender men (26% vs 14%, OR 1.97 (1.04–3.74), p = 0.010), and identify as MSW (34% vs 16%, OR 3.45 (1.79–6.72), p<0.001) or WSM (25% vs 15%, OR 2.81 (1.39–5.68), p<0.001) as compared to GBMSM. Between at least one MO vs zero MO groups, there were no differences by younger age groups, race/ethnicity, or proportion of individuals with an initial CD4 < 200 cells/ul. In the multivariable analysis, MSW and WSM activity (AOR 2.92 (1.46–5.87) and AOR 2.40 (1.15–4.98) respectively, p<0.004) as compared to GBMSM activity were the only significant predictors of MOs (Table 1).

**Table 1:** Factors predictive of having a missed opportunity for HIV screening among new HIV diagnoses 2018–2022.

	Missed HIV testing opportunities		Univariable				Multivariable		
	Zero, N = 169 (65%)^a^	One or more, N = 91 (35%)^a^	OR^b^	95% CI^b^	p	q^c^	AOR^b^	95% CI^b^	p
**Age group (y) **					**0.003**	**0.011**			0.2
*25 and younger *	34 (20%)	15 (16%)	—	—			—	—	
*25–49 *	117 (69%)	51 (56%)	0.99	0.50, 2.01			0.88	0.42, 1.91	
*50 and older *	18 (11%)	25 (27%)	3.15	1.35, 7.58			1.82	0.70, 4.82	
**Sex assigned at birth**					**0.035**	**0.061**			
*Male*	143 (84%)	67 (74%)	—	—					
*Female*	27 (16%)	24 (26%)	1.97	1.05, 3.69					
**Gender**					**0.010**	**0.024**			
*Cisgender man*	130 (77%)	66 (73%)	—	—					
*Cisgender woman*	24 (14%)	24 (26%)	1.97	1.04, 3.74					
*Transgender man*	2 (1.2%)	0 (0%)							
*Transgender woman*	9 (5.3%)	1 (1.1%)	0.22	0.01, 1.20					
*Gender non-conforming*	4 (2.4%)	0 (0%)							
**Race/ethnicity **					0.87	0.87			
*Hispanic *	80 (47%)	47 (52%)	—	—					
*Non-Hispanic Black *	74 (44%)	35 (38%)	0.81	0.47, 1.38					
*Non-Hispanic White *	12 (7.1%)	7 (7.7%)	0.99	0.35, 2.65					
*Other *	3 (1.8%)	2 (2.2%)	1.13	0.15, 7.08					
**Sexual activity** ^d^					**<0.001**	**0.001**			**0.004**
*GBMSM*	109 (69%)	34 (41%)	—	—			—	—	
*MSW *	26 (16%)	28 (34%)	3.45	1.79, 6.72			2.92	1.46, 5.87	
*WSM *	24 (15%)	21 (25%)	2.81	1.39, 5.68			2.40	1.15, 4.98	
**Intravenous drug usage ** ^**e**^	7 (4.1%)	3 (3.3%)	0.79	0.17, 2.91	0.73	0.85			
**Other risk factor ** ^**f**^	17 (10%)	9 (9.9%)							
**Unknown risk factor **	3 (1.8%)	6 (6.6%)							
**CD4<200 cells/ul** ^**g**^	38 (26%)	17 (22%)	0.80	0.41, 1.52	0.50	0.70			

^a^ n (vertical %).

^b^ OR = Odds Ratio, AOR = Adjusted Odds Ratio, CI = Confidence Interval.

^c^ False discovery rate correction for multiple testing.

^d^ Gay and bisexual men who have sex with men (GBMSM), men who have sex with women (MSW), women who have sex with men (WSM)

^e^ Reference group was individuals without a history of intravenous drug usage

^f^ Other risk factors included perinatal transmission, sexual assault, commercial sex work, having a partner living with HIV, and reported needlestick injury.

^g^ Reference group was individuals with an initial CD4>200 cells/ul. Some individuals reported multiple risk factors, (eg, homosexual sexual activity plus intravenous drug usage plus other risk factors) which were counted individually in each risk category (ie, not combined into only being counted for a single risk category).

We also examined whether any demographic, clinical or behavioral factors were associated with late diagnosis. In the univariable analysis, when compared to GBMSM activity, MSW but not WSM activity (OR 4.11 (1.97–8.66) and OR 2.27 (0.98, 5.11), respectively, p<0.001) was associated with a CD4 < 200 cells/ul at the time of diagnosis. The number of MOs (OR 0.97 (0.80–1.14), p = 0.69) was not associated with late diagnosis. In the multivariable analysis, when compared to GBMSM activity, MSW but not WSM activity remained a significant predictor of late diagnosis (AOR 3.65 (1.68–8.00) and AOR 2.04 (0.85–4.73) respectively, p = 0.004) (Table 2).

**Table 2:** Risk factors for late diagnosis defined as CD4 (cells/ul) <200 at the time of HIV diagnosis.

					Multivariable		
	OR^a^	95% CI^a^	p	q^b^	AOR^a^	95% CI^a^	p
**Age group (y) **			0.10	0.33			0.53
*25 and younger *	—	—			—	—	
*25–49 *	1.68	0.72, 4.40			1.56	0.62, 4.48	
*50 and older *	3.04	1.11, 9.02			1.90	0.61, 6.42	
**Sex assigned at birth**			0.59	0.80			
*Male*	—	—					
*Female*	1.23	0.57, 2.54					
**Gender**			0.50	0.80			
*Cisgender man*	—	—					
*Cisgender woman*	1.26	0.57, 2.62					
*Transgender man*	0.00						
*Transgender woman*	1.62	0.22, 8.62					
*Gender non-conforming*	0.00						
**Race/ethnicity **			0.98	0.98			
*Hispanic *	—	—					
*Non-Hispanic Black *	1.13	0.59, 2.15					
*Non-Hispanic White *	1.01	0.27, 3.14					
*Other *	0.82	0.04, 5.87					
**Sexual activity** ^d^			**<0.001**	**0.004**			**0.004**
*GBMSM*	—	—			—	—	
*MSW*	4.11	1.97, 8.66			3.65	1.68, 8.00	
*WSM*	2.27	0.98, 5.11			2.04	0.85, 4.73	
**Intravenous drug usage ** ^**c**^	1.94	0.39, 8.17	0.39	0.80			
**N missed opportunities in the previous year **	0.97	0.80, 1.14	0.69	0.80			

^a^ OR = Odds Ratio, AOR = Adjusted Odds Ratio, CI = Confidence Interval.

^b^ False discovery rate correction for multiple testing.

^c^ Reference group was individuals without a history of intravenous drug usage.

^d^ Gay and bisexual men who have sex with men (GBMSM), men who have sex with women (MSW), women who have sex with men (WSM)

Of the 228 total individuals with a reported CD4 count, 191 (83.8%) had a CD4 count result within 1 week of HIV diagnosis, 19 (8.3%) within 2–4 weeks, 16 (7.02%) within 1–3 months, and 2 (0.88%) within 3–6 months. Among those with zero vs at least 1 MO, there was no difference in the number of days between the date of the HIV diagnosis and the date of the first CD4 count (6.11±14.8 vs 10.9±25.9, p = 0.077). In addition, the number of missed opportunities did not correlate with the initial CD4 count (Pearson R (df = 226) = 0.0473, p = 0.48).

Using February 29, 2020 as the breakpoint, the average number of new HIV diagnoses per month was higher in the pre-COVID-19 era compared to the COVID-19 era (5.88±2.34 vs 3.82±1.83, p<0.001), coinciding with a greater number of total HIV tests performed per month in the pre-COVID-19 era (3762±339 vs 3017±598, p<0.001). We did not have access to the institutional data of the total number of healthcare system encounters by all individuals in the study period. However, among our cohort of newly diagnosed individuals, the number of healthcare system encounters (using the average number of MOs in the preceding 365 days per person as a surrogate) was similar during the pre-COVID-19 and COVID-19 eras (1.10±1.94 vs 1.00±2.00, p = 0.698). Demographic and clinical features between MO groups identified in the primary analysis remained similar overall when stratifying by pre-COVID-19 and COVID-19 eras (S1 Table). Of note, in both the COVID-19 and pre-COVID-19 eras, there was a greater proportion of GBMSM in the zero MO group and a greater proportion of MSW and WSM in the ≥ one MO group. The finding of older individuals being more likely to have a MO in the primary analysis appeared to be driven by the age distribution in the pre-COVID-19 era specifically (there was a greater proportion of individuals aged 50 years and older with one or more MO vs zero MO in the pre-COVID-19 era (28% vs 8%, p = 0.003) compared to the COVID-19 era (26% vs 14%, p = 0.2).

Of the 238 total MOs, 116 (48.7%) occurred in the ED, 109 (45.8%) in the outpatient setting (37 (15.5%) primary care, 59 (24.8%) subspecialty clinics, 13 (5.5%) OB/Gyn clinics), and 13 (5.5%) in the inpatient setting. Of the 59 MOs that occurred in subspecialty clinics, approximately one quarter had HIV screening offered but not performed. Compared to the outpatient setting, the ED and inpatient settings were less likely to document discussion of HIV prevention strategies (16%% vs 1% and 0%, p<0.001). Individuals were recorded as participating in HIV prevention discussions if there was any documentation of patient education regarding condom usage, PrEP, needle-exchange programs, or other HIV prevention strategies associated with the encounter in which HIV screening was not offered. The outpatient setting was more likely than the ED or inpatient setting to offer STI testing (4.6% vs 1.7% and 0%, p = 0.018), obtain a sexual history (29% vs 4.3% and 0%, p<0.001), and discuss condoms (6.4% vs 0% and 0%, p = 0.014) and PrEP (5.5% vs 0% and 0%, p = 0.030) (Table 3).

**Table 3:** Settings of missed HIV testing opportunities.

	ED, N = 116 (48.7%)^a^	Outpatient, N = 109 (45.8%)^a^	Inpatient, N = 13 (5.5%)^a^	p^b^
*Prior negative HIV test on record* ^c^	35 (30%)	45 (41%)	5 (38%)	0.2
*HIV testing offered *	30 (26%)	21 (19%)	3 (23%)	0.5
*STI testing offered *	2 (1.7%)	11 (10%)	0 (0%)	**0.018**
*STI testing performed *	2 (1.7%)	5 (4.6%)	0 (0%)	0.5
*Sexual history taken *	5 (4.3%)	32 (29%)	0 (0%)	**<0.001**
*Any HIV prevention strategies discussed *	1 (1%)	17 (16%)	0 (0%)	**<0.001**
*Safe sex discussed *	1 (0.9%)	5 (4.6%)	0 (0%)	0.2
*Condoms discussed *	0 (0%)	7 (6.4%)	0 (0%)	**0.014**
*PrEP discussed *	0 (0%)	6 (5.5%)	0 (0%)	**0.030**
*Already on PrEP *	0 (0%)	6 (5.5%)	0 (0%)	**0.030**

^a^ n missed opportunities (% of total missed opportunities in each setting).

^b^ Fisher’s exact test.

^c^ Prior negative HIV test on record indicated any negative HIV test done more than 365 days before the patient was diagnosed with HIV.

## Discussion

The purpose of this study was to identify demographic and clinical factors predictive of having (1) a MO for HIV screening and (2) a late diagnosis. Over one third of new diagnoses had at least one MO in the preceding year. Older individuals, cisgender women and those assigned female sex at birth, and heterosexual individuals were more likely to have at least one MO. These findings were overall similar when stratifying by pre-COVID-19 and COVID-19 eras. An initial CD4 < 200 cells/ul was more likely among MSW specifically. Most MOs occurred in the emergency department and outpatient settings, with minimal HIV prevention discussions documented during each MO.

Despite NYS-mandated HIV screening, 35% of newly diagnosed individuals at our large academic medical center in New York City had at least one MO during the study period, similar to the rate of 36% between 2006 and 2017 [11]. Although HIV testing rates decreased at our institution during the COVID-19 pandemic, the overall percentage of MOs has remained the same, suggesting persistent structural and knowledge gap barriers to screening [13].

Compared to GBMSM activity, MSW and WSM behavior was associated with having at least one MO and an initial CD4 of less than 200 cells/ul (while MSW activity was a significant predictor of late diagnosis, WSM activity only trended with late diagnosis). The differential likelihood of having MOs among GBMSM and MSW and WSM suggests that self-reported high-risk behaviors influence practitioner decisions to screen for HIV. Numerous provider survey studies have cited low risk perception as barriers to offering HIV screening [20]. Heterosexual individuals, specifically MSW, may have a lower self-perception of risk and consequently present for or request HIV screening at a lower rate [21]. Interestingly, provider-initiated HIV test offers have also been shown to be more prevalent among WSM than MSW [22, 23].

Cisgender women and individuals 50 years of age and older were more likely to have at least one MO. This is consistent with prior studies which identify women and older individuals as higher risk for late diagnosis [24, 25]. Overall, these misperceptions of risk on both the provider and individual level can lead to missed diagnoses and delayed initiation of antiretroviral therapy, which correlates with an increased risk of progression to AIDS and community transmission [2, 3].

The number of MOs was not associated with increased odds of a late diagnosis. This was likely due to a high degree of variability of CD4 counts by the number of MOs, and a relatively large proportion of individuals without any MOs being found to have a low initial CD4 count. As discussed in the limitations, encounters at medical centers outside of the study site were excluded from the analysis and thus the true number of MOs may have been underestimated. In addition, given the analysis only included MOs in the year preceding diagnosis, and that chronic HIV infection typically advances to AIDS in 10 years or longer, it is not surprising that the number of MOs in a single year preceding diagnosis did not predict late diagnosis [26].

The majority of those newly diagnosed with HIV identified as either Hispanic (49%) or non-Hispanic Black (42%). These groups of communities, who represent the majority of patients receiving care at our institution, have a disproportionately greater incidence of HIV in both New York State and nationally, and have disproportionately lower rates of PrEP coverage and viral suppression [27, 28].

Consistent with previous studies, the ED had a high rate of MOs [9, 10]. In nearly all MOs that occurred in the ED, there was no documentation of discussion regarding HIV screening, its potential benefits, and/or the easy availability of HIV prevention resources. While this was possibly due in part to the fast-paced and high-volume setting, other barriers likely contributed, such as insufficient privacy for counseling [29]. In the ED, HIV screening is more frequently performed among those who present during daytime hours, report HIV risk factors, and require venipuncture for other blood tests [30–33]. Although these factors are beyond the scope of this study, it is important to highlight the various negative and positive predictors of HIV screening in the ED to provide insight into the complexities of having MOs in the ED. In a recent survey, the vast majority of ED providers at our institution cited higher priority issues and time constraints as key barriers to HIV screening, underscoring the importance of prescreening questionnaires or nurse-driven screenings [15]. Considering the disproportionate burden of HIV among racial and ethnic minorities, many of whom are underinsured and utilize the ED as their primary source of medical care, expanding HIV screening is especially important in the ED [34].

In the outpatient setting, although providers were more likely to offer STI screening, obtain a sexual history, and discuss the use of condoms, MOs occurred at an overall similar rate to the ED and occurred most frequently in subspecialty clinics compared to primary care or OB/GYN clinics (the subspecialty clinic group in this study consisted of approximately 20 unique medical/surgical subspecialty, neurology, psychiatry, and dentistry clinics; data not shown). This highlights the need to incorporate routine HIV screening into all ambulatory encounters, perhaps with the aid of hard-stop clinical decision support in the electronic medical record if there is no evidence of a recent HIV test.

Given low risk perception is a widely cited patient and provider barrier to HIV screening, it is imperative to elicit detailed sexual histories either during the encounter or beforehand in the form of pre-visit questionnaires [20–23, 35]. Although to our knowledge there is not any literature demonstrating that sexual history documentation significantly correlates with reduced MOs, understanding patients’ risk for HIV acquisition is the first step in determining whether HIV screening is indicated. Thus, even framing an encounter as an opportunity for HIV screening requires a detailed sexual history. Future studies are needed to assess the impact of various methods of sexual history taking on expanding HIV screening and reducing MOs.

This study had several limitations. It is unclear how often HIV screening was offered and potentially declined, but not documented. Unfortunately, there was no electronic capture of HIV screening offers. Similarly, documentation of histories of sexual practices, substance use, and mental health disorders were often lacking, which impeded a full assessment of HIV risk factors for individuals. The true rate of MOs may have been underestimated considering encounters at other institutions were excluded from the analysis and individuals whose initial presentation to our institution for confirmatory HIV testing were considered new diagnoses if they did not report a prior diagnosis. The exact number of confirmatory patients who met criteria for MO was not recorded during data acquisition. Another limitation is that there was no process to assess reviewer agreement when extracting data from manual chart review.

## Conclusions

Despite NYS mandates for universal HIV screening, MOs have persisted at similar rates at our institution since 2006. MOs have occurred more frequently among populations perceived to be at lower risk, including older individuals, cisgender women, and heterosexual individuals. Implementing routine and regular HIV screening into the workflows of ED and ambulatory visits as well as inpatient admissions for all patients has the potential to improve early identification of new infections and subsequently reduce the risk of community transmission and disease progression [2, 3]. Ending the HIV epidemic will likely require greater attention to MOs in populations often considered at low risk for HIV.

## Supporting information

S1 Table Characteristics of newly diagnosed HIV patients with and without missed opportunities for testing in the previous year stratified by the COVID-19 pandemic timeline. (XLSX)

S1 Dataset (XLSX)
